# 

**Published:** 1857-03

**Authors:** 


No. X.] MARCH. [Vol. XII.

### BUFFALO MEDICAL JOURNAL

AND

### Monthly Review

OF

### MEDICAL AND SURGICAL SCIENCE.

SANFORD B. HUNT, M. D.,
EDITOR.

BUFFALO, N. Y.:

THOMAS & LATHROPS' STEAM PRESS.

Commercial Advertiser Buildings.

1857.

Price, $2.00 in advance, or $2.50 at the end of three months.

